# Development and Characterization of a New Oral Antileishmanial Bis(pyridine-2-Carboxamidine) Drug Through Innovative Dissolution Testing in Biorelevant Media Combined with Pharmacokinetic Studies

**DOI:** 10.3390/pharmaceutics17070838

**Published:** 2025-06-26

**Authors:** Almudena Laguna, Borja Martínez-Alonso, Víctor Guarnizo-Herrero, J. Jonathan Nué-Martinez, Christophe Dardonville, Santiago Torrado-Santiago, Carlos Torrado-Salmerón

**Affiliations:** 1 Department of Pharmaceutics and Food Technology, Faculty of Pharmacy, Complutense University of Madrid, Plaza Ramón y Cajal s/n, 28040 Madrid, Spain; almulagu@ucm.es; 2 Department of Biomedical Science, Faculty of Pharmacy, University of Alcalá de Henares, Ctra Madrid-Barcelona Km 33,600, 28805 Madrid, Spain; borja.martineza@uah.es (B.M.-A.); victor.guarnizo@uah.es (V.G.-H.); 3 Institute of Medicinal Chemistry, IQM-CSIC, Juan de la Cierva 3, 28006 Madrid, Spain; jonathan.nue@iqm.csic.es (J.J.N.-M.); dardonville@iqm.csic.es (C.D.); 4 University Institute of Industrial Pharmacy (IUFI), Complutense University of Madrid, 28040 Madrid, Spain

**Keywords:** antileishmanial drug, surfactant, characterization, biorelevant media, pharmacokinetic study

## Abstract

**Background/Objectives**: Currently there are very few effective oral antileishmanial treatments. In this study we evaluated a new bis(pyridine-2-carboxamidine) antileishmanial drug (JNII40_base) and its hydrochloride salt (JNII40_HCl). **Methods**: The characterization studies performed allowed us to determine the crystallinity, hydration water, and presence of hydrogen bonds in these drugs. Different dissolution methods were employed to predict intestinal absorption. A high-performance liquid chromatography–mass spectrophotometry (HPLC-MS/MS) method was developed for the determination of JNII40 in plasma. **Results**: Pharmacokinetic studies in rats of JNII40_base at 100 and 20 mg/kg, and JNII40_HCl at 20 mg/kg, showed a non-linear pharmacokinetic at high doses. An innovative biorelevant medium of phosphate buffer pH 6.8 with polysorbate 80 at 0.6% (*w*/*v*) showed high concentration values for JNII40_base at 30 min, which predicts good intestinal absorption. These results were consistent with the bioavailability data, which exhibited a significant (*p* < 0.05) increase in maximum plasma concentration (C_max_) and a slight delay in time to maximum (T_max_) compared to JNII40_HCl. Furthermore, the sustained release of JNII40_base in this biorelevant media was related to high plasma concentration values at 24 h (C_24h_) observed in bioavailability studies. These plasma concentrations of JNII40_base were above the half-maximal inhibitory concentration (IC_50_) against promastigote and amastigote forms of *Leishmania donovani*, which is indicative of effectiveness and should reduce the occurrence of drug resistance during treatments. **Conclusions**: The bioavailability and pharmacokinetic data support the consideration of this drug for further in vivo studies as an oral antileishmanial treatment.

## 1. Introduction

**Figure 1:**
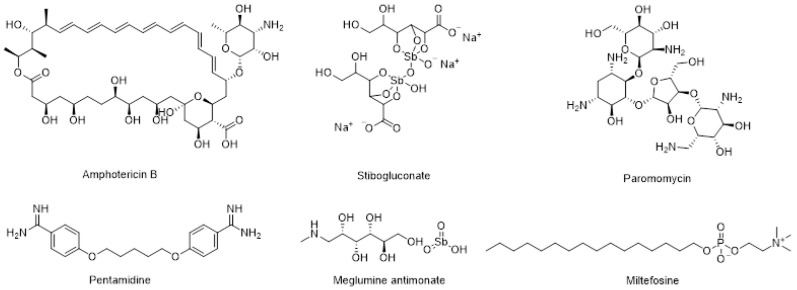
Structures of recommended drugs for VL and CL.

Leishmaniasis is a neglected tropical disease (NTD) that manifests primarily as cutaneous leishmaniasis (CL) caused by about 30 species of *Leishmania*, and visceral leishmaniasis (VL) caused by *Leishmania donovani* and *Leishmania infantum*. The incidence of this disease has increased in recent years due to environmental changes and increased human mobility from endemic areas [1,2]. Among the recommended drugs for leishmaniasis (Figure 1) [1], miltefosine is the only oral drug currently approved for the treatment of VL and CL [3,4,5]. However, its use is frequently associated with gastrointestinal side effects, and prolonged treatment can lead to significant liver toxicity [5,6]. Furthermore, recent reports on miltefosine have highlighted the emergence of unsatisfactory subclinical therapies [6] leading to an increase in miltefosine resistance in recent years [7,8]. Hence, new oral formulations of amphotericin B [9,10] or of alternative drugs such as buparvaquone [11] and artemisinin [12] have been studied. Additionally, new oral antileishmanial drugs that are both effective and safe are being developed [13,14].

**Figure 2:**
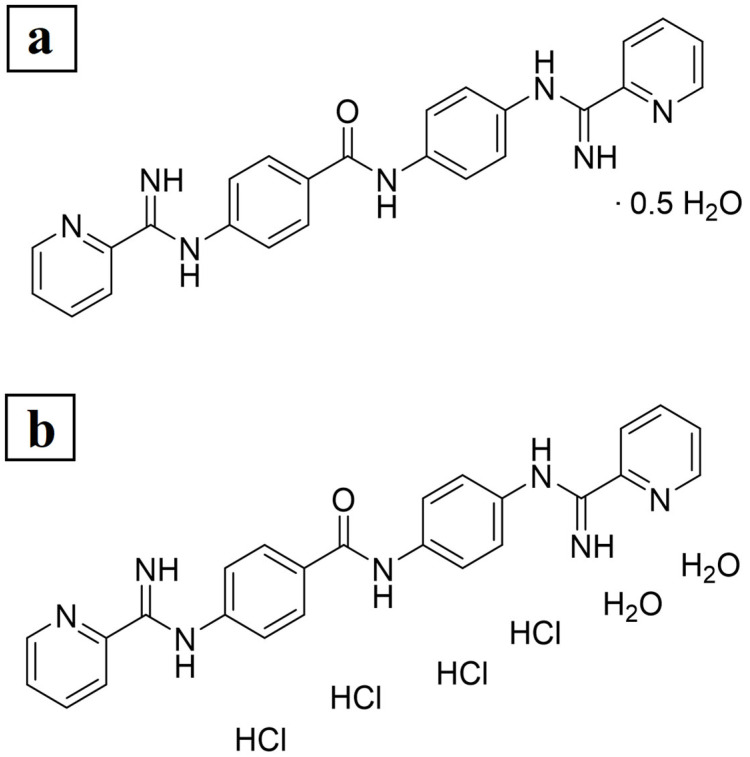
Elemental analysis. Chemical formula and experimentally determined content of (**a**) JNII40_base and (**b**) JNII40_HCl.

We recently reported a new antileishmanial compound, 4-picolinimidamido-N-[4-(picolinimidamido)phenyl]benzamide (JNII40_base), that showed potent efficacy in vitro against *L. donovani*, with IC_50_ values of 0.26 ± 0.05 µM and 0.65 ± 0.20 µM against promastigote and intracellular amastigotes of *L. donovani*, respectively (Figure 2) [15]. This compound, which proved to be stable towards hepatic metabolism and in human serum, is a promising oral treatment candidate for in vivo studies in animal models of VL. However, JNII40_base exhibited low kinetic solubility in water at pH 7.4, with a value of 6.35 ± 0.63 µM (2.8 ± 0.3 µg/mL), which may preclude in vivo oral bioavailability.

The use of salts and salt-crystal hydrates is a strategy to improve the solubility and dissolution of poorly soluble drugs [16,17,18]. Different salt forms can lead to significant changes in plasma concentration–time profiles and key pharmacokinetic parameters after oral absorption [18,19]. In fact, the hydrochloride salt of the drug (JNII40_HCl) demonstrated significantly higher water solubility (194 ± 19 µM; 120 ± 12 µg/mL at pH 7.4). However, predicting in vivo oral bioavailability using biorelevant dissolution media is an important step to understand the in vivo performance of drugs.

Table 1 provides a summary of reported biorelevant dissolution media that are used to predict oral absorption. Biorelevant media such as Fasted State Simulated Gastric Fluid (FaSSGF) and Fasted State Simulated Intestinal Fluid (FaSSIF) exhibit supersaturation processes [20,21]. For certain poorly soluble drugs, biorelevant media that show both supersaturation and precipitation processes are more effective in predicting oral absorption [22,23,24]. Increases in dissolution profiles in these media have been attributed to the presence of bile acids and improved wettability of poorly soluble drugs [24]. Other biorelevant media contain various surfactants, such as sodium lauryl sulfate (SDS) or polysorbate 80. The use of low concentrations of SDS (<0.25%) and polysorbate 80 (<0.3%) has resulted in biorelevant media that maintain surfactant levels below their critical micelle concentration [25,26,27,28]. In these cases, supersaturation is observed without further precipitation processes. In media with low proportions of SDS [20,27] and polysorbate 80, wettability improvement has been identified as the primary mechanism for increased drug concentrations [29,30,31]. However, when high concentrations of SDS (0.5%) and polysorbate 80 (0.5%) are used, significant changes are observed [20,32,33]; in particular, the presence of micellar systems in the media leads to high supersaturation concentrations followed by precipitation [20,32]. On the other hand, biorelevant media provide a means to track changes in crystallinity during precipitation processes, revealing crystal growth that follows various crystallization kinetics [22,23].

These literature findings suggest that adjusting surfactant proportions in the biorelevant medium should enhance the wettability of JNII40_base and JNII40_HCl, and may prevent precipitation and recrystallization of the drug from a supersaturated state [20]. In this work, we investigated different biorelevant dissolution media to identify the most suitable one for predicting in vivo absorption and oral bioavailability parameters of JNII40_base and JNII40_HCl salt. This study is useful for the selection of formulations that should be used in pharmacokinetics with healthy animals after repeated doses.

We conducted various characterization techniques, such as elemental analysis (EA), differential scanning calorimetry (DSC), Fourier transform infrared spectroscopy (FTIR), and scanning electron microscopy (SEM), to explore the physicochemical properties of JNII40_base and JNII40_HCl salt. The HPLC-MS/MS method developed and validated in this study was found to be suitable for the quantification of JNII40 in plasma samples. Finally, the study of different pharmacokinetic parameters that relate plasma concentrations to in vitro efficacy values (IC_50_) was important for the selection of oral doses that can prevent the appearance of possible resistance to *Leishmania* spp.

## 2. Materials and Methods

### 2.1. Materials

Polysorbate 80 (Tween^®^ 80), acetonitrile, methanol, sodium hydroxide, sodium acetate 3-hydrate, hydrochloric acid 37% *v*/*v*, and formic acid were obtained from Panreac (Barcelona, Spain). Sodium carboxymethyl cellulose was obtained from Mehta Pharmaceutical (Barcelona, Spain). Water was ultra-pure (Milli-Q) and, as well as the rest of the reagents and chemicals used, was laboratory grade.

### 2.2. Methods

#### 2.2.1. Preparation of JNII40_base and JNII40_HCl Formulations

The JNII40_base and JNII40_HCl compounds’ salt forms were used for solid state characterization analysis (EA, DSC, FTIR and SEM). The process of synthesis to obtain this new antileishmanial drug JNII40_base has been described previously [15].

Formation of 4-picolinimidamido-*N*-[4-(picolinimidamido)phenyl]benzamide tetrahydrochloride salt (JNII40_HCl): Compound JNII40_base (4.6 g) was suspended in water and methanol. A concentrated aqueous solution of hydrochloric acid (35%) was added slowly to the stirred mixture until complete dissolution of the product. The pH of the solution was checked (pH < 3), and excess hydrochloric acid was removed by rotary evaporation using a water jet vacuum pump. The remaining solvents were then removed by rotary evaporation under vacuum (membrane pump) to yield JNII40_HCl as a yellow solid (quantitative). Compounds JNII40_base and JNII40_HCl were characterized by ^1^H NMR, LC-MS (Supplementary Figures S1 and S2), and HPLC (UV) > 98% purity.

JNII40_base and JNII40_HCl formulations were prepared by adding 50 mg of JNII40_base or JNII40_HCl to 3 mL of sodium carboxymethylcellulose solution (0.50% *w*/*v*). The suspension was stirred at 2000 rpm for 2 min. These suspensions were employed as formulations for dissolution studies. To prepare oral suspensions for animal studies, 316.05 mg of JNII40_base was suspended with 4 mL of sodium carboxymethylcellulose solution (0.50% *w*/*v*) at 2500 rpm for 2 min to administrate a dose of 100 mg/kg of JNII40_base. For oral administrations of 20 mg/kg of JNII40_base and JNII40_HCl, 63.20 mg of JNII40_base and JNII40_HCl, respectively, was suspended with 4 mL of sodium carboxymethylcellulose solution (0.50% *w*/*v*) at 2500 rpm for 2 min.

#### 2.2.2. Solid State Characterization

##### Elemental Analysis (EA)

Elemental analyses (C, H, N) of JNII40_base and JNII40_HCl were performed after drying under vacuum at room temperature for three days. A comparison of the calculated values and the experimental results was made.

##### Differential Scanning Calorimetry (DSC)

A TA DSC25 instrument (TA Instruments, New Castle, DE, USA) was used to analyze the thermal properties of the samples. The temperature of the instrument was standardized using the indium reference material. Precisely weighed samples were loaded into hermetically sealed aluminum pans and heated from 25 °C to 350 °C at a constant rate of 10 °C/min under continuous flushing of dry nitrogen at a flow rate of 20 mL/min. An empty sealed pan was utilized as a reference to account for any background thermal effects.

##### Fourier Transform Infrared Study (FTIR)

A Frontier FTIR Spectrometer applying universal attenuated total reflection (PerkinElmer, Waltham, MA, USA) was used to perform Fourier transform infrared (FTIR) spectroscopy. For sample preparation, 2.5 mg of JNII40_base or JNII40_HCl was weighed. The scanning range was 400–4000 cm^−1^ with a spectral resolution of 4 cm^−1^.

##### Scanning Electron Microscopy (SEM)

The samples were mounted on an aluminum sample mount. After coating with a thin layer of gold–palladium, the hydrogel samples were analyzed via SEM using a Jeol^®^ IT700. All micrographs were the product of secondary electron imaging used for surface morphology identification at different magnifications and an accelerating voltage of 5 kV. JNII40_base and JNII40_HCl actives were mounted and analyzed. Oven dried samples of JNII40_base and JNII40_HCl dissolution studies in phosphate buffer pH 6.8 and polysorbate 80 (0.6%) medium were also scanned with SEM.

#### 2.2.3. Dissolution Studies

JNII40_base and JNII40_HCl were evaluated in Fasted State Simulated Intestinal Fluid (FaSSIF) medium (pH 6.5) and phosphate buffer (pH 6.8) at a concentration of 0.1%, 0.3%, 0.6%, and 0.8% of polysorbate 80 mediums under non-sink conditions. The dissolution studies were performed at 10 mL of each medium, 75 rpm, and 37.0 ± 0.5 °C using a Multimatic 5-S magnetic stirrer with a thermostatic bath (J. P. Selecta; Barcelona, Spain). An amount of 5 mg of JNII40_base or JNII40_HCl salt was added to 10 mL of each medium. This amount of drug and the volume of dissolution medium were selected for an adequate study of supersaturation and precipitation [31]. A sample of 1 mL was withdrawn and filtered through a 0.45 µm filter (Syringe filter, PES, 25 mm, 0.45 µm, Agilent Technologies, Santa Clara, CA, USA) with volume replacement at 5, 10, 15, 20, 30, 45, 60, 90, and 120 min. The filtered samples were immediately diluted, and the quantity of active pharmaceutical ingredient was determined at 274 nm using a UV-VIS spectrophotometer (Jasco Analitica S.L.; Madrid, Spain). The cumulative amount of JNII40_base or JNII40_HCl salt was determined from the following calibration line: y = 60495x + 5.146 (R^2^ = 0.9997) across a range of 2.5–20 µg/mL. Each determination at each time was performed in triplicate and the error bars on the graphs represent the standard deviation (SD).

#### 2.2.4. Solubility Studies

Solubility studies were performed with an excess of JNII40_base and JNII40_HCl samples added to different test tubes containing 3 mL of pH 6.8 phosphate buffer (pH 6.8) at a concentration of 0, 0.1, 0.3, 0.6, and 0.8% (*w*/*v*) of polysorbate 80 medium. These samples were mixed in a vortex and shaken in a water bath at 37 °C for 48 h. Each sample was filtered through a 0.45 μm filter (Acrodisc^®^, Port Washington, NY, USA), diluted, and analyzed at 274 nm using a UV-VIS spectrophotometer (Jasco Analitica S.L.; Madrid, Spain). The cumulative amount of JNII40_base or JNII40_HCl salt was determined from the following calibration line: y = 60495x + 5.146 (R^2^ = 0.9997) across a range of 2.5–20 µg/mL. Each determination at each time was performed in triplicate.

#### 2.2.5. Animal Study

This experiment was performed with 18 male Wistar rats supplied by Envigo Rms Co., Ltd. (Barcelona, Spain), with an average weight of 316.71 ± 8.79 g upon arrival. The animals were housed at the Animal Experimentation Center at the University of Alcalá de Henares. This study was carried out following the rules of the Ethics Committee of the University of Alcalá de Henares and was approved by the Community of Madrid with the project identification code PROEX 369.7/21 (20 December 2021). Animals were housed in standard cages with controlled room temperature (22–24 °C) and 12 h light/dark cycles, and unrestricted access to food and water throughout the experiment.

#### 2.2.6. Plasma Concentration-Time Profile

The pharmacokinetic studies were carried out in three groups of animals (*n* = 6). Animals were fasted overnight before the experiment. Treatments of 100 mg/kg of JNII40_base, 20 mg/kg of JNII40_base, and 20 mg/kg of JNII40_HCl were suspended in 0.4 mL of sodium carboxymethylcellulose solution (0.50% *w*/*v*) and administered to the rats through oral gavage. Blood samples were collected in heparinized Eppendorf tubes at 0.5, 1, 2, 4, 6, 8, and 24 h. The samples were centrifuged at 4000 rpm for 10 min to collect the plasma. The plasma homogenates were stored at −20 °C.

#### 2.2.7. HPLC-MS/MS Analytical Method for Pharmacokinetics

Plasma samples were analyzed using a HPLC-MS/MS mass spectrometer. High-performance liquid chromatography (HPLC) was performed on an Agilent 1100 series apparatus (Agilent, Waldbroon, Germany). The compound was separated using a Kinetex F5 (2.6 μm × 2.1 mm × 100 mm) column. A binary gradient elution using mobile phases containing water in mobile phase A (0.1% formic acid) and acetonitrile (0.1% formic acid) in mobile phase B was used with a flow rate of 0.3 mL/min and an injection volume of 5 μL at a temperature of 22 °C. The gradient program was followed as follows: 0–10.0 min, 80% A and 20% B; 10.0–15.0 min, 25% A and 75% B; and 15.0–19.0 min, 80% A and 20% B. All components were eluted in 19 min.

The MS-MS detection was carried out using a Thermo TSQ Quantum LC/MS Triple Quadrupole instrument (Waltham, MA USA) equipped with an electrospray (ESI) source in positive mode and detection was performed in multiple reaction monitoring (MRM) mode. The following conditions were used to configure the mass spectrometry ion source as positive mode ESI: capillary temperature, 300 °C; sheath gas pressure, 40 L/h; and capillary voltage, 3000 V. MRM mode with positive ions was used for quantifying the transition, and the detection of the ions was performed in MRM mode, monitoring the transition of the *m*/*z* 436.2 precursor ion [M + H]^+^ to the *m*/*z* 419.2 product ion for JNII40, and 315.1 and 224.1 product ions as qualifier ions. The collision energy was 24 V, 31 V, and 43 V respectively.

Plasma samples for analysis were obtained from blood samples after centrifugation at 4000 rpm for 10 min. The purification process was performed with pre-columns (Phree^™^ Phospholipid, Phenomenex, Torrance, CA, USA). Aliquots of 0.150 mL of plasma were added with 0.450 mL of acetonitrile solution. The loaded pre-columns were placed in a vacuum manifold system for solid-phase extraction (SPE) at 15 inches Hg for 5 min. The final product was filtered through a 0.20 µm PTFE Millex^™^ LG filter.

A calibration curve was constructed for JNII40 standard solutions. Blank plasma samples were added to a working analyte standard solution at a plasma:standard ratio of 9:1 *v*/*v*. A calibration curve was obtained as the average of three injections of three calibration points, showing regression coefficients R^2^ > 0.9991 within the linearity range between 15 and 300 ng/mL (*n* = 9). This method exhibited a limit of detection (LOD) of 1.5 ng/mL and a limit of quantification (LOQ) of 15.0 ng/mL. In addition, the repeatability, accuracy, and recovery of the extraction were evaluated, presenting good results according to the analytical methods of the European Medicines Agency [36].

#### 2.2.8. Bioavailability Parameters

Non-compartmental pharmacokinetic parameters were obtained for individual rats in each group from plasma concentration–time curve data within 24 h of drug administration [36]. The maximum plasma concentration (C_max_), plasma concentration at 24 h (C_24h_), and time to reach C_max_ (T_max_) were estimated as the mean values of the six animals used per group with SD. The areas under curves from time zero to 24 h plasma concentration (AUC_0–24h_) were calculated by means of the trapezoidal rule. Comparative statistical studies on the bioavailability of the different formulations were performed using Tukey’s test, with a one-way analysis of variance (ANOVA) test in the Statgraphics program (Statgraphics Technologies, The Plains, VA, USA).

#### 2.2.9. Statistics Analysis

Differences between the obtained values (mean ± standard error) for the different samples were explored using a one-way ANOVA, followed by an appropriate post hoc test in the case of the presence of a significant difference. A *p*-value of less than 0.001 or 0.05 was considered a criterion for a statistically significant difference (Statgraphics Plus, version 5.1).

## 3. Results and Discussion

### 3.1. Characterization at the Solid State

#### 3.1.1. Elemental Analysis

The results of the elemental analysis for compound JNII40_base are shown in Figure 2a. The calculated content for C_25_H_21_N_7_O/0.5 H_2_O was C, 67.55; H, 4.99; N, 22.06, while the experimentally determined content was C, 67.61; H, 5.46; N, 21.60. According to the elemental analysis, JNII40_base is partially hydrated and contains 0.5 molecules of H_2_O, which likely accounts for the hygroscopic nature of the compound.

The elemental analysis of compound JNII40_HCl showed a calculated content of C_25_H_21_N_7_O/4 HCl/2 H_2_O (MW = 617.35): C, 48.64; H, 4.74; N, 15.88, while the experimentally determined content was C, 48.63; H, 4.77; N, 15.85. According to the elemental analysis, JNII40_HCl is a tetrahydrochloride salt and is hydrated with 2 molecules of H_2_O (see Figure 2b). This is consistent with the method of preparation of this salt form using an excess of aqueous hydrochloric acid. In addition, the ^1^H NMR data of JNII40_HCl (Supplementary Figure S2) are consistent with these results because 29 H are visible in the aromatic region (7.5–12.5 ppm) of the spectrum (i.e., 16 ArH, 9 NH, and 2 H_2_O). The similarity between the calculated and experimental content confirms the high purity of the compound, as well as the different water contents in each form [37].

#### 3.1.2. Differential Scanning Calorimetry (DSC) and Fourier Transform Infrared Study (FTIR)

**Figure 3:**
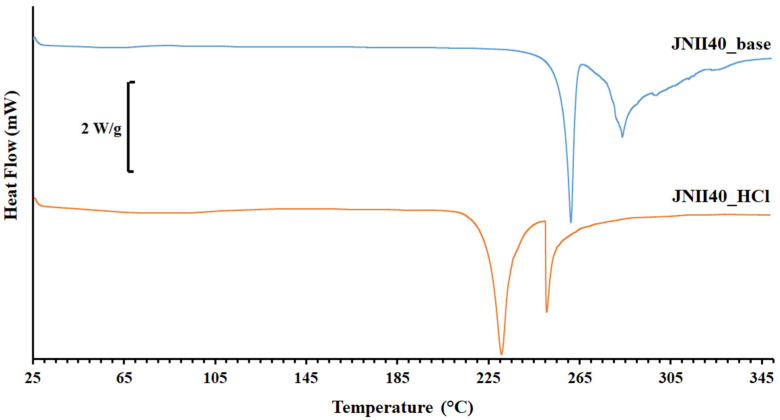
Thermograms of differential scanning calorimetry (DSC) of JNII40_base and JNII40_HCl.

Figure 3 shows the DSC scan of JNII40_base, which exhibits a possible glass transition (Tg) at ~60 °C. The absence of endothermic changes around ~100 °C confirms a low presence of water molecules in the structure, previously observed in the elemental analysis (Figure 2). A first sharp endothermic peak was observed at 260.25 °C. The high enthalpy value ∆H 161.92 J/g was associated with a crystalline form. A second thermal event occurred as a broad endothermic peak at 282.57 °C, which was attributed to a degradation process [38].

Ionic hydrochloride salts (e.g., JNII40_HCl) can form thermodynamically stable structures that promote drug supersaturation. Unfortunately, these salts can also accelerate drug precipitation [39]. The DSC scan of JNII40_HCl showed a broad endothermic peak between 70 and 105 °C, which was attributed to the presence of the two water molecules observed in the elemental analysis [38,40]. This endothermic event prevented the observation of the enthalpic relaxation (Tg) of the JNII40_HCl molecule. A shift to lower temperatures was observed for the first endothermic peak (231.22 °C, and ∆H 71.01 J/g). This suggests that, after dehydration, the salt is predominantly crystalline, and the water molecules within the hydrated form are not part of the crystal packing arrangement. However, significant changes were observed in the second endothermic peak, with a sharp melting peak at low temperature and higher enthalpy values (251.15 °C, and ∆H 174.88 J/g). This second sharp endothermic peak was associated with the hydrochloride salt form [38]. The significant reduction in the melting point values indicates a decrease in the crystal lattice energy [36], which may have a positive impact on the dissolution profile [41,42].

**Figure 4:**
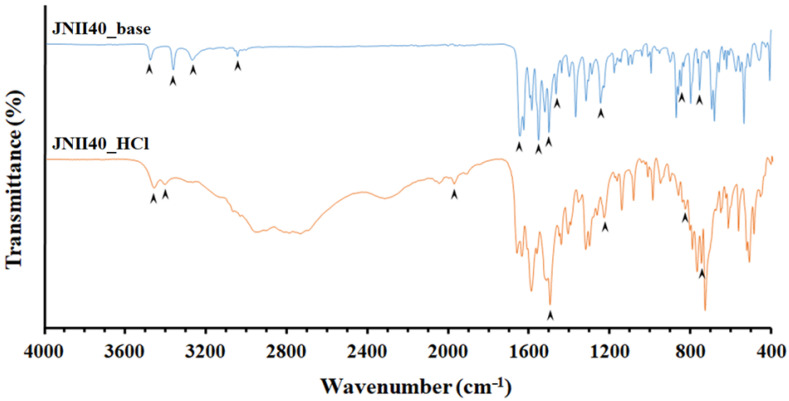
Fourier transform infrared spectroscopy (FTIR) spectra of JNII40_base and JNII40_HCl.

The FTIR spectrum (Figure 4) of JNII40_base showed characteristic bands at 3551, 3369, and 3274 cm^−1^, corresponding to primary amine N–H stretch vibrations. Similar amine N–H stretch vibrations bands have been previously described by several authors in amphotericin and chitosan molecules; these bands were related to an increase in wettability with water molecules of the dissolution medium [43,44]. The band at 3050 cm^−1^ was attributed to aromatic =CH stretching vibrations [45]. The peak at 1647 cm^−1^ was assigned to –C=N or –C=0 stretching vibration groups, while the band at 1555 cm^−1^ was assigned to the N–H stretching vibration of amine groups [44]. The peak at 1504 cm^−1^ was attributed to the vibration of the benzene ring [46]. The band at 1468 cm^−1^ was assigned to the C–H alkane group, and peaks at 848 and 755 cm^−1^ were attributed to C=C alkene groups. Finally, the peak at 1247 cm^−1^ was attributed to C–N stretching [8,42].

During the formation process of the hydrochloride salt (JNII40_HCl), a transformation in the crystallinity was evident. The regions between wavelengths 3551 and 3274 cm^−1^ showed a disappearance of several vibration bands, and only two bands at 3477 and 3412 cm^−1^ were observed that could be attributed to an interaction with water molecules [39]. Under these conditions, the amino groups are protonated (i.e., positively charged), and an interaction between the N–H groups and water molecules present in the dehydrated form of JNII40_HCl was observed. Similar changes were noted in other amphoteric drugs, such as amphotericin B, which can be protonated or deprotonated in an aqueous solution depending on the pH [10].

Additionally, a new band appeared at 1968 cm^−1^ due to the presence of hydrogen bonds (O···H–N), which was related to a water uptake process during salt formation. This band coincides with the absence of the band at 1555 cm^−1^, which is attributed to the N–H stretching vibration of amine groups [44]. The peak at 1497 cm^−1^ was attributed to the vibration of the benzene ring [46]. The remaining vibration bands did not show any significant changes. Peaks at 835 and 754 cm^−1^ were attributed to the C=C alkene bond. The peak corresponding to 1238.90 cm^−1^ was attributed to the stretching of C–N [9,47].

#### 3.1.3. Scanning Electron Microscopy (SEM) Characterization

**Figure 5:**
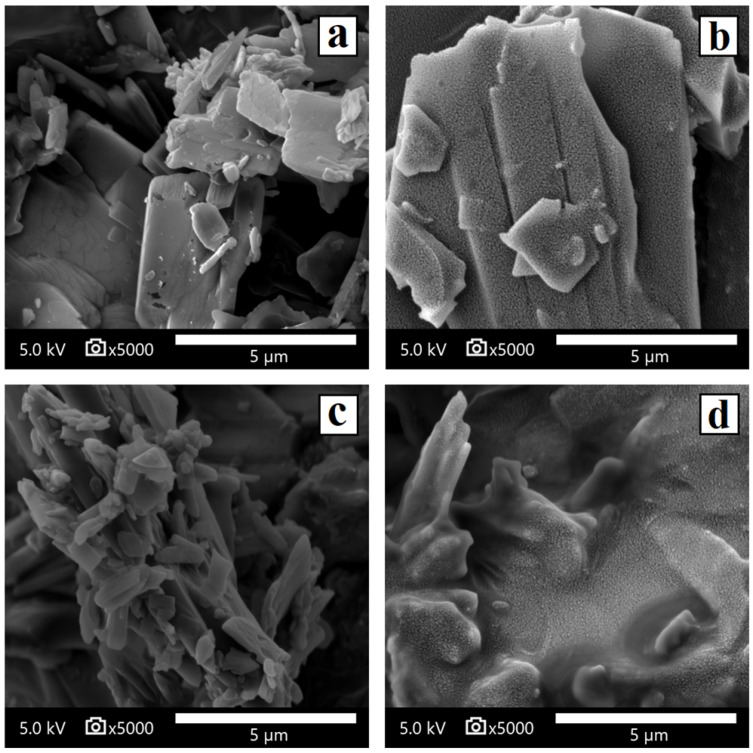
Scanning electron microscopy micrographs of surface-modified (**a**) JNII40_base, (**b**) JNII40_HCl, (**c**) JNII40_base in biorelevant media with polysorbate 80 (0.6%), and (**d**) JNII40_HCl in biorelevant media with polysorbate 80 (0.6%). Photographs were taken at a magnification of 5000×.

Scanning electron microscopy of JNII40_base showed small crystalline orthorhombic structures with a smooth surface and a particle size between 2 and 5 µm (Figure 5a), while JNII40_HCl showed small crystalline orthorhombic structures with a particle size of 5–10 µm (Figure 5b).

The SEM micrographs of JNII40_base in phosphate buffer pH 6.8 with polysorbate 80 (0.6%) showed significant morphological changes on the surface of the particles, with a partial smooth coating attributed to the polysorbate 80 film around the JNII40_base crystals (Figure 5c). The high wettability of polysorbate will increase the dissolution rate of the JNII40_base crystals [36]. In contrast, the SEM images of JNII40_HCl crystals in phosphate buffer pH 6.8 with polysorbate 80 (0.6% *w*/*v*) exhibited important changes, with polysorbate 80 forming a rough film that interacts with the JNII40_HCl crystals and covers the surface of the crystalline particles (Figure 5d). The interactions observed in the FTIR studies of JNII40_HCl could be related to these important changes observed in biorelevant media with 0.6% polysorbate 80. The presence of the surfactant film decreases the crystallinity of JNII40_HCl and increases its wettability in aqueous media. Similar SEM observations when evaluating possible changes in morphology during dissolution studies have been reported previously [22,48].

### 3.2. Dissolution Studies

**Figure 6:**
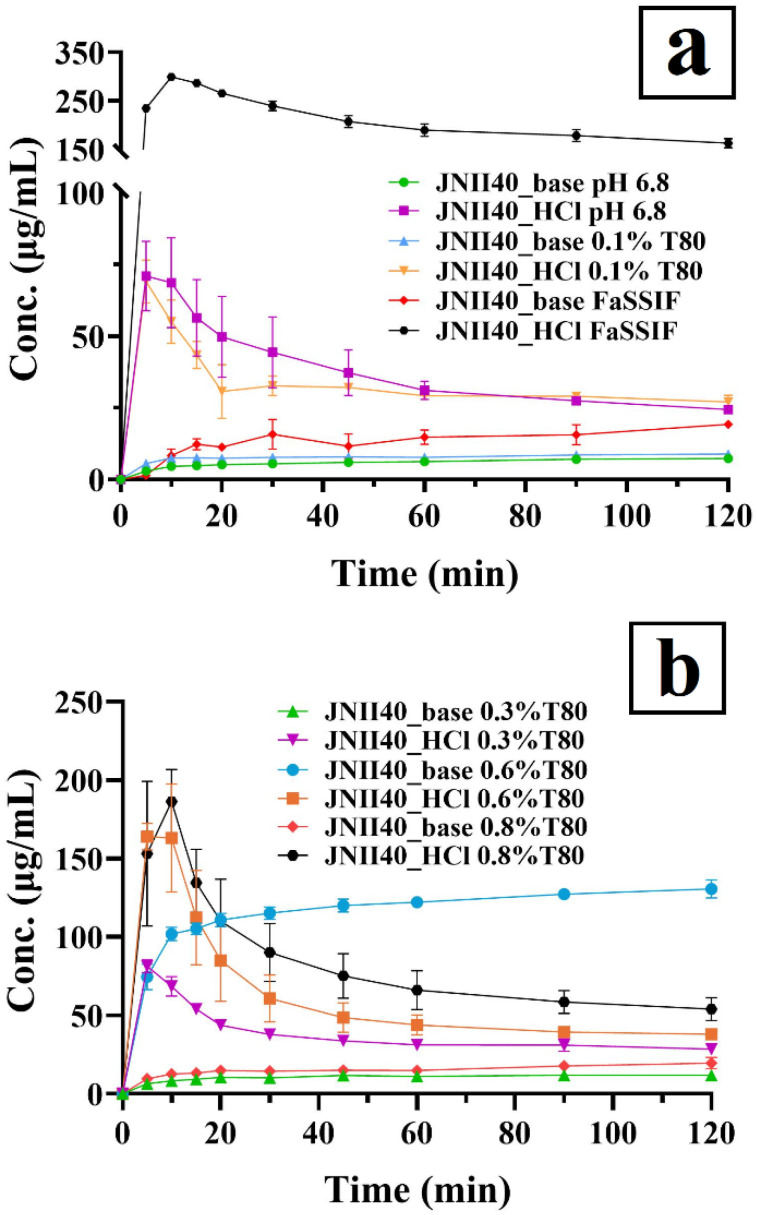
Dissolution profiles of JNII40_base and JNII40_HCl in different media. (**a**) Phosphate buffer (pH 6.8), FaSSIF (pH 6.5) and phosphate buffer (pH 6.8) with 0.1% polysorbate 80. (**b**) Phosphate buffer (pH 6.8) with 0.3% polysorbate 80, phosphate buffer (pH 6.8) with 0.6% polysorbate 80, and phosphate buffer (pH 6.8) with 0.8% polysorbate 80. Mean ± standard deviation (SD) (*n* = 3).

The dissolution profiles of JNII40_base and JNII40_HCl in phosphate buffer (pH 6.8) and biorelevant FaSSIF medium (pH 6.5) are shown in Figure 6a. Dissolution studies with different percentages of polysorbate 80 (0.3, 0.6, and 0.8%) are shown in Figure 6b. This study was conducted to select the percentage of surfactant that allowed us to obtain discriminative averages for each drug [26,31].

#### 3.2.1. Dissolution Studies in Different Intestinal Simulated Media

The salt JNII40_HCl in phosphate buffer (pH 6.8) exhibited a low release profile, with a concentration of 68.68 ± 15.73 µg/mL at 10 min. This was followed by a slight precipitation process starting at 10 min, which resulted in concentrations of approximately 24.40 ± 0.10 µg/mL at 120 min (Figure 6a). The high hydrophilicity of JNII40_HCl compared to JNII40_base and its greater wettability with the dissolution medium may be attributed to the presence of hydrogen bonds and water molecules, as observed in FTIR studies for JNII40_HCl. Increased wettability facilitates water interaction on the surface, enhancing dissolution [32,34,49]. In contrast, JNII40_base displayed significantly lower dissolution profiles in phosphate buffer (pH 6.8), with dissolution concentration at 10 min of 4.61 ± 1.03 µg/mL and 7.34 ± 0.22 µg/mL at 120 min (*p* < 0.001). These lower dissolution rates can be attributed to its reduced solubility. During the dissolution study of JNII40_base, its high hydrophobicity led to the formation of large aggregates, which floated on the dissolution medium. Hydrophobic drugs tend to reduce surface area, thereby delaying dissolution [34,50]. Similar low dissolution profiles in intestinal media have been observed with other antiparasitic drugs, such as arteether, an artemisinin derivative [30]. In the case of poorly soluble drugs, the low release profiles observed in phosphate buffer (pH 6.8) underscore the importance of using alternative media, such as FaSSIF (pH 6.5), or buffers incorporating low concentrations of surfactants, such as polysorbate 80 (0.1% *w*/*v*) added to the phosphate buffer (pH 6.8) [24,29].

In a biorelevant FaSSIF medium (pH 6.5), JNII40_HCl exhibited a high dissolution profile, with significant increases (*p* < 0.001) of 4.35-fold at 10 min and 6.66-fold at 120 min, compared with the non-biorelevant medium (i.e., phosphate buffer pH 6.8). These results suggest that bile salts and phospholipids form micellar structures, leading to substantial improvements in the dissolution profile of JNII40_HCl [24,50]. However, JNII40_base in FaSSIF medium exhibited a low increase of 1.80-fold and 2.62-fold at 10 min and 120 min, compared to the non-biorelevant medium (phosphate buffer pH 6.8). The greater hydrophobic character of JNII40_base resulted in the formation of aggregates on the surface of the dissolution medium, hindering solubilization by bile salts and resulting in smaller increases in the dissolution profiles [21,50]. However, the dissolution profiles in FaSSIF (pH 6.5) do not fully explain the differences observed in the pharmacokinetic profiles between JNII40_HCl and JNII40_base.

The dissolution profiles of JNII40_base and JNII40_HCl in phosphate buffer (pH 6.8) containing polysorbate 80 (0.1% *v*/*v*) are shown in Figure 6a. In this medium, JNII40_HCl exhibited a rapid initial dissolution of 55.02 ± 7.60 µg/mL at 10 min and a steady state until 120 min (27.07 ± 2.29 µg/mL). The high initial dissolution rate can be attributed to a significant increase in wettability. The low concentration of polysorbate 80 (0.1% *w*/*v*) is below its critical micellar concentration in the dissolution medium, preventing the formation of micelles [25,26]. Nevertheless, the presence of surfactant in the dissolution medium results in slight increases in the dissolution profiles of JNII40_base, with concentrations of 7.47 ± 0.12 µg/mL at 10 min and 8.87 ± 0.21 µg/mL at 120 min. These results suggest that polysorbate 80 at a concentration of 0.1% in the dissolution medium is insufficient to form micellar systems [50]. However, the presence of low concentrations of surfactants improves wettability on the surface of the hydrophobic drug, enhancing molecular interaction with the dissolution medium [26].

#### 3.2.2. Dissolution Studies in Biorelevant Media at Different Concentrations of Polysorbate 80

Dissolution studies at pH 6.8 in biorelevant media with different proportions of surfactant (0.3%, 0.6%, and 0.8%) provide insights into supersaturation and precipitation processes. Biorelevant media with higher concentrations of polysorbate 80 are associated with elevated levels of bile salt and lecithin micelles, which are present in the proximal segments of the small intestine. In contrast, reduced supersaturation and increased precipitation can be expected in the distal regions of the intestine, where lower levels of bile salts and lecithin micelles are present [51,52].

The dissolution of JNII40_HCl with low proportions of polysorbate 80 (0.3% *w*/*v*) in phosphate buffer (pH 6.8) produced similar concentration values at 10 min (1.00-fold) and 120 min (1.17-fold) compared to concentrations obtained in phosphate buffer without surfactant (Figure 6b). Moreover, a significant precipitation process was observed, resulting in a 2.85-fold decrease in concentration after 2 h (see Figure 6b). Previous work indicated that the critical micelle concentration of polysorbate 80 was 0.3%, so this low percentage of surfactant allowed only a low number of micelles in the dissolution medium [25,26]. The low proportion of micelles in the medium was not able to improve the dissolution of JNII40_HCl [50]. However, a higher release profile was observed for JNII40_base in a medium with a low surfactant percentage (0.3% *w*/*v*). High increases (*p* < 0.001) of 1.78-fold at 10 min and 1.62-fold at 120 min compared to concentrations obtained in phosphate buffer (pH 6.8) without surfactant were observed (Figure 6b). These findings suggest that a low surfactant concentration improves wettability and enhances the dissolution profile of hydrophobic drugs such as JNII40_base [29]. Similar improvements in dissolution profiles for poorly soluble drugs have been observed in surfactant media without a micellization process [32].

Increasing the percentage of polysorbate 80 surfactant in phosphate buffer (pH 6.8) to 0.6% (*w*/*v*) induced a high number of micelles in the dissolution medium. In this medium (Figure 6b), JNII40_HCl produced a significant supersaturation process, with increments of 2.37-fold at 10 min compared to JNII40_HCl in phosphate buffer (pH 6.8). However, a significant precipitation process was observed between 15 and 45 min. Similar supersaturation and precipitation processes for poorly soluble drugs were observed with different micellar media [24,30]. Different behavior was observed for JNII40_base in phosphate buffer (pH 6.8) with 0.6% polysorbate 80. In this biorelevant medium, JNII40_base showed a significant increase (*p* < 0.001) of 22.08-fold and 17.78-fold at 10 min and 120 min, respectively, compared to JNII40_base in phosphate buffer (pH 6.8) without surfactant (Figure 6b). The sustained release profile and the absence of a precipitation process was related to a significant presence of micelles in the dissolution medium [24,32].

Finally, the study of biorelevant media with high proportions of polysorbate 80 (0.8%) in phosphate buffer (pH 6.8) showed changes in the dissolution profiles of JNII40_HCl and JNII40_base (see Figure 6b). In this medium, JNII40_HCl produced an important supersaturation process, with increases (*p* < 0.001) of 2.71-fold at 10 min compared to JNII40_HCl in phosphate buffer (pH 6.8). This was followed by a drastic precipitation process between 10 and 45 min. Between 45 min and 2 h, a slow precipitation process was observed, with a significant (*p* < 0.001) 2.21-fold increase in concentration at 120 min compared to phosphate buffer (pH 6.8) without surfactant [24]. Unlike what would be expected, different behavior was observed for JNII40_base in phosphate buffer (pH 6.8) with 0.8% (*w*/*v*) polysorbate 80. In this medium, JNII40_base displayed a slow dissolution profile showing only slight increases of 2.73-fold and 2.66-fold at 10 min and 120 min, respectively, compared to phosphate buffer (pH 6.8) without surfactant. The high percentage of surfactant in the medium produced a less soluble polysorbate salt with JNII40_base and did not allow the formation of the supersaturation process [32].

### 3.3. Solubility Studies in Biorelevant Media: Influence of Ionic Strength and Polysorbate 80 Proportions

**Figure 7:**
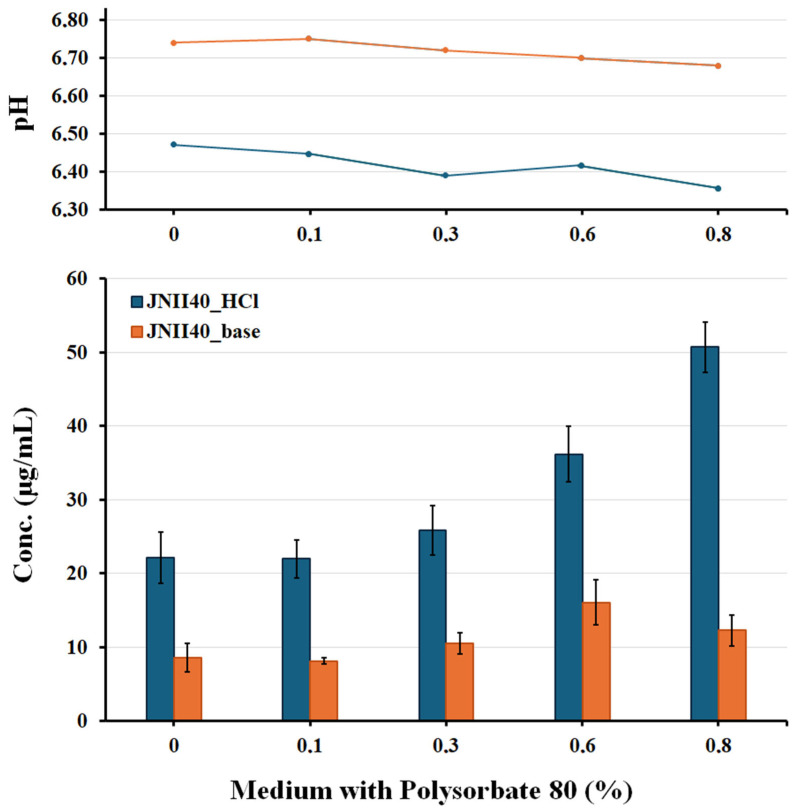
pH and solubility studies of JNII40_base and JNII40_HCl in biorelevant media. Influence of ionic strength and different proportions [0, 0.1, 0.3, 0.6, and 0.8% (*w*/*v*)] of polysorbate 80.

The solubility study with media at pH 6.8 exhibited high concentrations of JNII40_HCl (22.14 ± 3.426 µg/mL), which are similar to the dissolution values observed at 2 h in Figure 6a. This elevated solubility of salts with strong acids (hydrochloric acid) in low ionic strength media may be related to a “self-buffering effect” [53]. In the dissolution and solubility studies, hydronium ions (H_3_O^+^) appeared; these ions are represented by the decrease in pH values (6.47 ± 0.052) and the release of Cl^−^ in the presence of the salt (Figure 7) in this medium.

Solubility studies with phosphate buffer at pH 6.8 and low concentrations of polysorbate 80 (0.1% and 0.3%) showed low concentrations of JNII40_HCl (21.98 ± 2.619 and 25.88 ± 3.317 µg/mL) at 48 h (Figure 7). The low surfactant content can enhance crystal growth and drug precipitation processes. These media with a low proportion of surfactant also presented a pH reduction that confirms the presence of hydronium ions (H_3_O^+^) and suggests a self-buffering effect.

Phosphate buffer at pH 6.8 with high concentrations of polysorbate 80 (0.6% and 0.8%) showed significant increases (*p* < 0.001) in solubility at 48 h, with concentrations of 36.18 ± 3.762 and 50.72 ± 3.367 µg/mL. The presence of micelles in the solubility medium increases hydronium ions (H_3_O^+^) and has a self-buffering effect, as confirmed by the decrease in the pH of the medium (Figure 7). Similar increases in concentration values were previously observed in dissolution studies at 2 h; these results were attributed to the presence of micellar structures that improve solubility. Dissolution and solubility studies showing comparable increases caused by the presence of surfactants have been reported previously for poorly soluble ionizable drugs [50].

JNII40_base exhibited different behavior during the solubility studies. Solubility tests with a phosphate buffer at pH 6.8 without surfactant showed concentrations of 8.63 ± 1.90 µg/mL at 48 h (Figure 7). These concentrations were similar to the dissolution values obtained at 2 h (Figure 6a). The poor solubility of weakly basic drugs has previously been associated with low dissolution profiles in biorelevant media [54].

Solubility studies of JNII40_base in phosphate buffer at pH 6.8 containing low concentrations of polysorbate 80 (0.1% and 0.3%) showed a low solubility (8.17 ± 0.41 and 10.52 ± 1.459 µg/mL), similar to the concentrations presented at 2 h in the dissolution studies (Figure 6a,b). These data indicate that micellar structures could not be formed in these conditions, and the low surfactant concentrations can increase the precipitation of JNII40_base [30,32]. The pH values between 6.72 and 6.75 confirmed the lack of hydronium ions (H_3_O^+^) and the decrease in the solubility of JNII40_base compared to its salt form.

The higher concentrations of polysorbate (0.6% and 0.8%) in the phosphate buffer at pH 6.8 led to a significant increase (*p* < 0.001) in the solubility values of JNII40_base at 48 h, with an increase of 1.97- and 1.50-fold respectively, compared to the solubility obtained with 0.1% polysorbate 80. This enhancement was attributed to micelle formation in these media [26,28]. The solubility results with 0.6% polysorbate suggest that precipitation begins after 2 h in the dissolution studies. Similar dissolution profiles, involving sustained supersaturation followed by precipitation, have been reported previously [30,32].

To sum up, JNII40_HCl in a biorelevant medium at pH 6.8 with 0.3% and 0.6% polysorbate showed a high initial solubility, followed by a precipitation process after 2 h (see Figure 6a). Solubility studies confirmed the precipitation observed in the dissolution profiles. These supersaturation and precipitation results could be indicative of a potential in vivo precipitation process [52].

Different behavior was observed for JNII40_base in biorelevant medium at pH 6.8 containing 0.3%, 0.6%, and 0.8% polysorbate 80, i.e., sustained release with a supersaturation process during the dissolution study. The high surfactant concentrations in the biorelevant medium simulate the presence of elevated levels of bile salts and lecithin micelles in the proximal small intestine [52].

### 3.4. Pharmacokinetic Study

Several reports have explored the relationship between pharmacokinetic studies and the efficacy of different oral antileishmanial treatments using poorly soluble drugs, such as miltefosine and buparvaquone [5,11]. The promising results obtained from the in vitro studies of JNII40 against different kinetoplastid parasites encouraged us to conduct an in vivo pharmacokinetic study in healthy rats following a single oral dose [15,55].

The developed method proved suitable for the rapid determination of JNII40_base in plasma samples (retention time 0.80 min) (Supplementary Figure S3). It showed good linearity, with a LOD of 0.5 ng/mL and a LOQ of 5.0 ng/mL, which are appropriate for the detection of low concentrations of JNII40 in plasma samples. The analytical method validation demonstrated good repeatability (≤4.89%), accuracy (≤4.94%), and recovery (≤6.55%). The results of this validation study (Supplementary Table S1) confirmed that the method is suitable for the quantification of JNII40.

**Figure 8:**
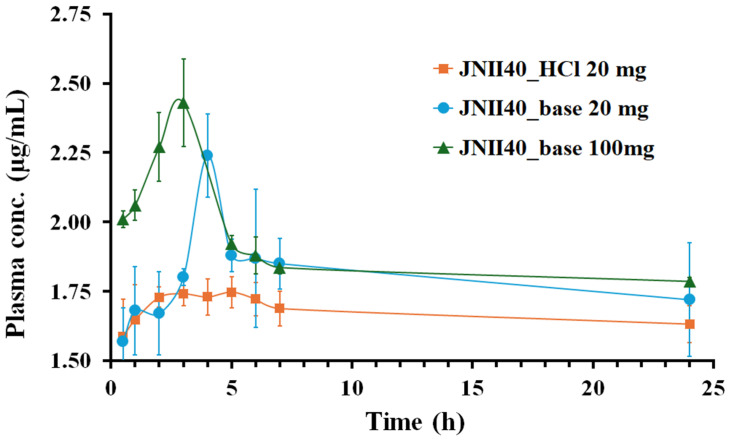
Plasma concentration profile of JNII40_base at 100 and 20 mg/kg and JNII40_HCl at 20 mg/kg. Mean ± SD (*n* = 6).

The pharmacokinetic behavior of the different formulations (JNII40_base and JNII40_HCl) was evaluated in male Wistar rats. The plasma concentration–time curves of JNII40 following the oral administration of JNII40_base at doses of 100 and 20 mg/kg, and JNII40_HCl at 20 mg/kg, are shown in Figure 8.

The pharmacokinetic studies revealed an early onset of action for both JNII40_base and JNII40_HCl, with similar, non-significant (*p* > 0.05) plasma concentrations observed at 0.5 h for both formulations at 20 mg/kg. However, distinct concentration–time profiles were observed between JNII40_base and JNII40_HCl formulations.

The JNII40_base formulation at 100 and 20 mg/kg exhibited a high concentration–time peak, with C_max_ between 3 and 4 h, followed by a significant decrease between 4 and 7 h. These results are consistent with the supersaturation profiles observed between 0 and 30 min in biorelevant media (pH 6.8 with polysorbate 80 at 0.3% and 0.6%). The decrease in plasma concentrations may be attributed to a precipitation process, as well as the low solubility values obtained in these biorelevant media. In contrast, the JNII40_HCl formulation did not exhibit a concentration–time peak, which was likely due to the high precipitation observed at 10 min in the biorelevant intestinal media (pH 6.8 with polysorbate 80 at 0.3%, 0.6%, and 0.8%). Similar precipitation and poor absorption behavior have been reported in previous studies with buparvaquone SNEDDS for oral antileishmanial treatment [11].

The high-dose (100 mg/kg) of JNII40_base formulation demonstrated the highest plasma concentrations, peaking between one and seven hours. In contrast, the low doses (20 mg/kg) of JNII40_base formulation showed a slight delay, with a concentration peak occurring between two and seven hours. However, the JNII40_HCl formulation did not exhibit this characteristic peak (Figure 8). This could be attributed to the significant precipitation observed for the JNII40 salt form in the dissolution studies in biorelevant media of phosphate buffer with polysorbate 80, which may have hindered the typical concentration–time profile. Previous studies have indicated that poorly soluble drugs often experience precipitation in the gastrointestinal tract, which can influence their pharmacokinetic profiles [11].

The mean pharmacokinetic parameters (C_max_, T_max_, C_24h_, and AUC_0–24h_) for the three groups are summarized in Table 2.

The C_max_ and AUC_0–24h_ values for JNII40_base at 100 and 20 mg/kg did not show significant differences (*p* > 0.05) (Table 2), and only a slight increase in bioavailability (108.77 ± 3.14%) at a dose of 100 mg/kg compared to JNII40_base at 20 mg/kg was observed. The limited increase in bioavailability at higher doses suggests non-linear pharmacokinetics [56,57]. The poor aqueous solubility of the compound likely contributes to the observed non-linear pharmacokinetic behavior. Similar saturable absorption has been observed for miltefosine [6]. Furthermore, analysis of plasma samples revealed high protein binding for JNII40_base, whose partition coefficient (log *p*) is 2.76, suggesting a high lipid affinity. However, plasma protein binding is not necessarily a disadvantage for an antileishmanial drug because it could increase its accumulation in the target organs, such as the liver and spleen [5].

The pharmacokinetic comparison of JNII40_base and JNII40_HCl at 20 mg/kg revealed significant improvements (*p* < 0.05) of 1.28-fold in the C_max_ for JNII40_base, with no significant differences (*p* > 0.05) observed in C_24h_ and AUC_0–24h_ parameters. The results also indicated a slight decrease in bioavailability (94.20 ± 3.69%) for JNII40_HCl compared to JNII40_base. The rapid dissolution profile of JNII40_HCl formulation likely explains the slightly lower T_max_ (*p* > 0.05) compared to JNII40_base (Table 2). Finally, biorelevant media with different proportions of polysorbate 80 proved adequate to predict different “in vivo” absorption parameters [11,58]. The high C_24h_ values observed for both JNII40_base and JNII40_HCl formulations suggest a potential cumulative effect after repeated doses, a phenomenon previously reported for oral miltefosine formulations [5].

The phenomenon of drug resistance to the existing treatments against *Leishmania* spp. is a problem that needs to be considered early in the drug development process [59]. Earlier studies have shown that being able to maintain high exposure times to plasma drug concentrations several times higher than the IC_50_ values (t > *n* × IC_50_) is important to reduce the appearance of resistance to treatments [7]. Table 3 shows, for JNII40_base formulations at 100 and 20 mg/kg, the number of times that the plasma concentrations C_max_ and C_24h_ are above the in vitro IC_50_ values obtained for this drug in promastigotes and intracellular amastigotes of *L. donovani* [15]. In recent studies investigating the relationship between plasma and target organ concentrations and effectiveness against promastigote and amastigote forms (IC_50_ and IC_90_ values), it was found that a reduction of more than 75% in promastigote growth was observed at concentrations of 2 × IC_50_ [8]. In addition, a significant decrease in drug resistance has been reported with treatments at concentrations > 2 × IC_50_ and >8 × IC_50_ [5,8].

The results in Table 3 indicate that the *n* × IC_50_ values for C_24h_ are similar between the 100 and 20 mg/kg doses of JNII40_base. The results of non-linear pharmacokinetics and their similar concentration/efficacy results allow us to select the 20 mg/kg dose as the most appropriate, with C_24h_ values of up to 15 × IC_50_ and 6 × IC_50_ for promastigotes and amastigotes, respectively [15]. In previous studies of efficacy for different *Leishmania* spp. and pharmacokinetics in rodents, a reduction in promastigote growth of more than 75% has been observed with concentrations 2 × IC_50_ and 8 × IC_50_ [5,8]. The JNII40_base formulation showed plasma concentrations up to six times above the IC_50_ (t > 6 × IC_50_) and was maintained for more than 24 and 21 h for promastigote and amastigote, respectively. In addition, the high half-life values (t_1/2_ > 200 h) observed for this drug allow us to expect increases in these plasma values after repeated doses, which should limit the appearance of drug resistance.

These results allow us to consider JNII40_base at 20 mg/kg as a suitable formulation for future efficacy studies at repeated doses in a murine model of leishmaniasis [5,7,60].

## 4. Conclusions

The physicochemical properties of this novel antileishmanial drug and its hydrochloric salt, along with the use of different biorelevant media, were assessed to predict the differences in in vivo absorption observed in pharmacokinetic studies following a single dose of either JNII40_base or JNII40_HCl salt.

Characterization techniques such as EA, DSC, FTIR, and SEM revealed that JNII40_HCl exhibited higher hydration compared to JNII40_base, attributed to a greater number of hydrogen bonds, which facilitated its dissolution and precipitation processes in biorelevant media.

A series of studies were conducted using biorelevant media in phosphate buffer (pH 6.8) with different proportions of polysorbate 80 (0.1, 0.3, 0.6, and 0.8%). In these biorelevant media, JNII40_HCl presented a supersaturation phase, followed by precipitation. In contrast, the JNII40_base form exhibited a sustained release profile, without any precipitation during the dissolution in the biorelevant medium.

Bioavailability studies of JNII40_base at 100 and 20 mg/kg, as well as JNII40_HCl salt at 20 mg/kg, indicated that the new drug is safe and exhibits a non-linear pharmacokinetics. In vivo studies with JNII40_base exhibited a significant increase in C_max_ and a slight delay in T_max_ compared to JNII40_HCl. Furthermore, the plasma concentration of JNII40 exceeds the IC_50_ values obtained in vitro for this compound several-fold. These results encourage us to conduct further oral studies with repeated doses of JNII40_base to evaluate its safety profile and accumulation in target organs following repeated administration.

## Figures and Tables

**Table 1:** Composition of dissolution studies of different oral formulations in biorelevant media.

Drug/Formulation	Media	Supersaturation Time (Increase-Fold)	Process	Ref.
Dipyridamole Ketoconazole	FaSSIF ^1^ pH 6.5	Supersaturation 30 min; Precipitation dipyridamole 60–120 min and ketoconazole 30–60 min	Precipitation was related to a crystallization process	Higashino et al., 2023 [23]
Buparvaquone SNEDDS ^3^	Lipolysis medium pH 6.5 bile salts and phosphatidylcholine	Supersaturation 60–80 min; No precipitation process	Bile salts and lipase suspension promote Lypolysis of SNEDDS	Smith et al., 2018 [11]
Dipyridamole	Two state FaSSGF ^2^ to FaSSIF ^1^	Supersaturation 20 min; Precipitation 20–180 min	Precipitation was related to a crystallization process	Arnold et al., 2011 [22]
Telmisartan Solid dispersion	1.5% SDS ^6^ in phosphate buffer pH 7.4	Supersaturation 20–60 min ~3.5–4.0-fold; No precipitation process	Increase wettability and available surface area	Aldeeb et al., 2022 [34]
Arteether SLN ^4^ (2% polysorbate 80)	0.1% polysorbate 80 in PBS pH 7.4	Supersaturation at 50 min ~1.7-fold; No precipitation process	Increase wettability and available surface area	Dwivedi et al., 2014 [30]
Niterdipine micronized tablets (2.5% SDS ^6^)	DSPS ^5^ (Krebs-Ringer buffer pH 6.8)	Supersaturation at 30 min ~1.17-fold; Precipitation 30–240 min	Permeability dependent on pre-dissolved drug solution	Chen et al., 2023 [29]
Drug B tablets (poorly soluble cationic drug)	0.5% SDS ^6^ in sodium acetate buffer pH 4.5	Supersaturation at 60 min ~4.0-fold; No precipitation process	Micelle facilitated dissolution; Less soluble dodecyl sulfate salt of Drug B	Huang et al., 2018 [32]
Ritonavir	FaSSIF ^1^ pH 6.8	Supersaturation at 30 min Precipitation 30–60 min	Bile acid increases solubilization	Naing et al., 2024 [24]
Albendazole:Soluplus 1:5 Solid dispersions (P407 5%)	FaSSIF ^1^	Supersaturation 45–90 min ~2.0-fold; No precipitation process	Improve wettability and prevent drug recrystallization	Saha et al., 2023 [20]
Docetaxel granules (Mygliol 812: TPGS 1:1)	0.5% polysorbate 80 PBS pH 7.4	Supersaturation 10–240 min ~2.5-fold; No precipitation process	Micelle-facilitated dissolution	Shah et al., 2022 [26]
ODM-106 nanosuspension (0.1% SDS ^6^)	0.5% polysorbate 80 phosphate buffer pH 6.8 *non-sink* condition	Supersaturation 0–30 min >2-fold; Precipitation process 60–60 min	Polysorbate 80 increase wettability	Singhal et al., 2022 [27]
Felodipine Co-amorphous systems	0.25% SDS ^6^ in water solution	Supersaturation 30–60 min 1.53~1.87-fold; No precipitation process	Amorphization increased wettability and available surface area	Li et al., 2021 [35]

^1^ FaSSIF: Fasted State Simulated Intestinal Fluid; ^2^ FaSSGF: Fasted State Simulated Gastric Fluid; ^3^ SNEDDS: Self-Nanoemulsion Drug Delivery System; ^4^ SLN: Solid lipid nanoparticle; ^5^ DSPS: Dissolution-perfusion system; ^6^ SDS: Sodium lauryl sulfate.

**Table 2:** Pharmacokinetic parameters maximum plasma concentration (C_max_), time to reach C_max_ (T_max_), plasma concentration at 24 h (C_24h_), and areas under curves from time zero to 24 h plasma concentration (AUC_0–24h_) for JNII40_base at 100 and 20 mg/kg and JNII40_HCl at 20 mg/kg. Mean ± standard deviation (SD) with *n* = 5.

Plasma Parameters	JNII40_Base 100 mg/kg	JNII40_Base 20 mg/kg	JNII40_HCl 20 mg/kg
C_max_ (µg/mL)	2.42 ± 0.26	2.24 ± 0.22	1.75 ± 0.15
T_max_ (h)	3.00 ± 0.00	4.00 ± 0.00	3.40 ± 0.89
C_24h_ (µg/mL)	1.77 ± 0.23	1.68 ± 0.18	1.63 ± 0.22
AUC_0–24h_ (µg h/mL)	44.68 ± 3.68	41.69 ± 4.29	39.32 ± 5.02

**Table 3:** Pharmacokinetics-pharmacodynamics parameters C_max_, C_24h_ and t > 6 × IC_50_ * for JNII40_base at 100 and 20 mg/kg. Mean ± SD with *n* = 5.

Plasma Parameters	JNII40_Base 100 mg/kg	JNII40_Base 20 mg/kg
Promastigote		
C_max_	21.4 × IC_50_	19.8 × IC_50_
C_24h_	15.7 × IC_50_	14.9 × IC_50_
t > 6 × IC_50_	24 h	24 h
Amastigote		
C_max_	8.5 × IC_50_	7.9 × IC_50_
C_24h_	6.3 × IC_50_	6.0 × IC_50_
t > 6 × IC_50_	23.5 h	21 h

* IC_50_ values against promastigotes and intracellular amastigotes of *L. donovani* [15].

## Data Availability

The raw data supporting the conclusions of this article will be made available by the authors on request.

## Supplementary Materials

The following supporting information can be downloaded at: https://www.mdpi.com/article/10.3390/pharmaceutics17070838/s1, Figure S1: 1H NMR spectra and HPLC-MS traces of JN-II40_base; Figure S2: 1H NMR spectra and HPLC-MS traces of JN-II40_HCl; Figure S3: Representative HPLC-MS/MS chromatograms of JNII40 showing (a) standard sample at 15 ng/mL (LLOQ), (b) standard sample at 150 ng/mL, (c) plasma sample at low concentration, and (d) plasma sample at medium concentration.; Table S1: HPLC-MS/MS assay performance data for JNII40. Repeatability, accuracy, and SPE extraction recovery were established at three concentrations: 15 ng/mL (Low), 150 ng/mL (Medium), and 300 ng/mL (High). Each run contained six replicates per concentration tested.

## Abbreviations

The following abbreviations are used in this manuscript:

NTD	Neglected tropical disease
CL	Cutaneous leishmaniasis
VL	Visceral leishmaniasis
IC_50_	Inhibitory concentration 50
IC_90_	Inhibitory concentration 90
JNII40_base	Name of the base compound: 4-picolinimidamido-N-[4-(picolinimidamido)phenyl]benzamide
JNII40_HCl	Hydrochloride salt form of the JNII40 compound
FaSSGF	Fasted State Simulated Gastric Fluid
FaSSIF	Fasted State Simulated Intestinal Fluid
SDS	Sodium lauryl sulfate
SNEDDS	Self-Nanoemulsifying Drug Delivery Systems
PBS	Phosphate buffered saline
SLN	Solid lipid nanoparticle
DSPS	Dissolution-perfusion system
P407	Poloxamer 407
TPGS	Tocopherol Polyethylene Glycol Succinate
EA	Elemental analysis
DSC	Differential scanning calorimetry
FTIR	Fourier transform infrared spectroscopy
SEM	Scanning electron microscopy
HPLC-MS/MS	High-performance liquid chromatography–mass spectrometry
UV-VIS	Ultraviolet–visible spectrophotometry
^1^H NMR	Proton nuclear magnetic resonance
LC-MS	Liquid chromatography–mass spectrometry
MRM	Multiple reaction monitoring
ESI	Electrospray ionization
SPE	Solid-phase extraction
LOD	Limit of detection
LOQ	Limit of quantification
C_max_	Maximum plasma concentration
T_max_	Time to maximum plasma concentration
AUC_0–24h_	Area under the curve from 0 to 24 h
ANOVA	Analysis of variance
SE	Standard error
Tg	Glass transition
SD	Standard deviation
C_24h_	Plasma concentration at 24 h
Log *p*	Partition coefficient
t_1/2_	Half-life
