# Supplementary material for: Development and Characterization of a New Oral Antileishmanial Bis(pyridine-2-Carboxamidine) Drug Through Innovative Dissolution Testing in Biorelevant Media Combined with Pharmacokinetic Studies
Source: Pharmaceutics. 2025 Jun 26;17(7):838. doi: 10.3390/pharmaceutics17070838 (PMC12300650; doi:10.3390/pharmaceutics17070838)
Supplement: Supplementary file 1 [file pharmaceutics-17-00838-s001.zip › pharmaceutics-3669237-supplementary.pdf]

Supplementary Material

Table S1. HPLC-MS/MS assay performance data for JNII40. Repeatability, accuracy, and SPE extraction recovery were established at three concentrations: 15 ng/mL (Low), 150 ng/mL (Medium), and 300 ng/mL (High). Each run contained six replicates per concentration tested.

	Repeatability (n=6)		Accuracy (n=6)		SPE extraction recovery (n=6)	
**Spiked Conc (ng/mL)**	Mean % (range)	RSD (%)	Mean % (range)	RSD (%)	Mean (%) (range)	RSD (%)
15	103.97% (98.67 – 114.00)	4.89%	104.10% (98.67 – 114.00)	4.94%	95.00% (92.33 – 106.67)	5.43%
150	100.92% (94.67 – 103.80)	3.42%	101.33% (96.00 – 105.33)	3.29%	92.54% (84.47 – 98.67)	5.79%
300	101.71% (97.33 – 105.67)	3.15%	101.14% (96.67 – 105.33)	2.90%	92.99% (85.67 – 95.33)	6.55%

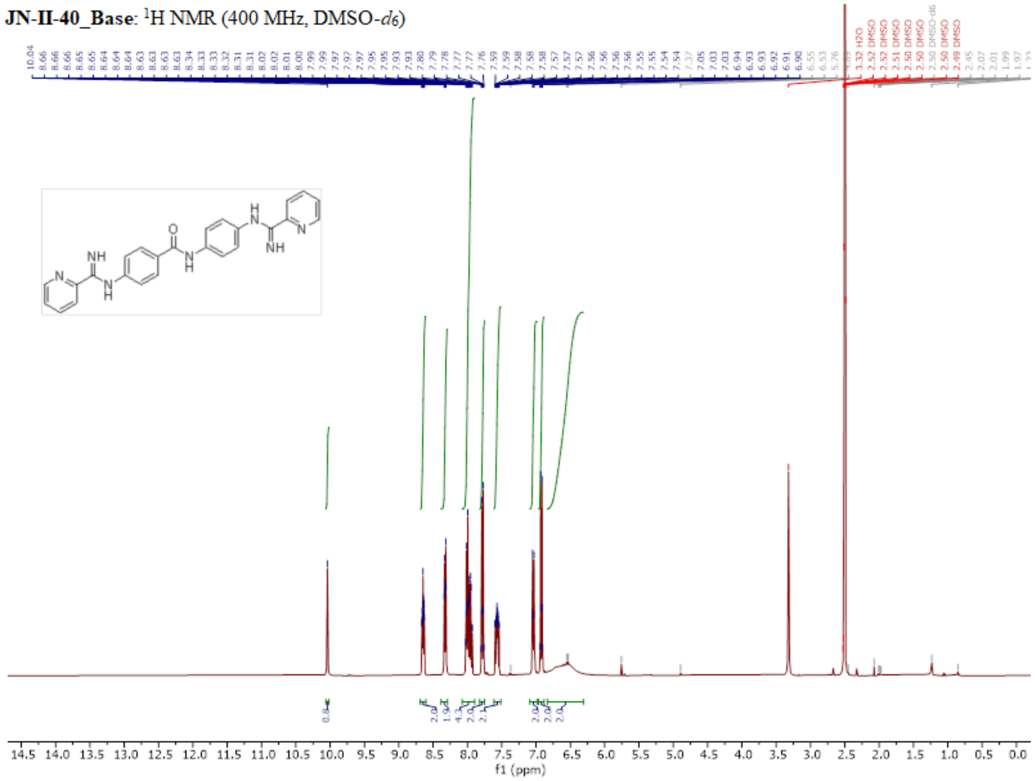

Supplement Figure 1. ^{1}H NMR spectra and HPLC-MS traces of JN-II40_base

JN-II-40_HCl: ^{1}H NMR (400 MHz, DMSO-*d6*)

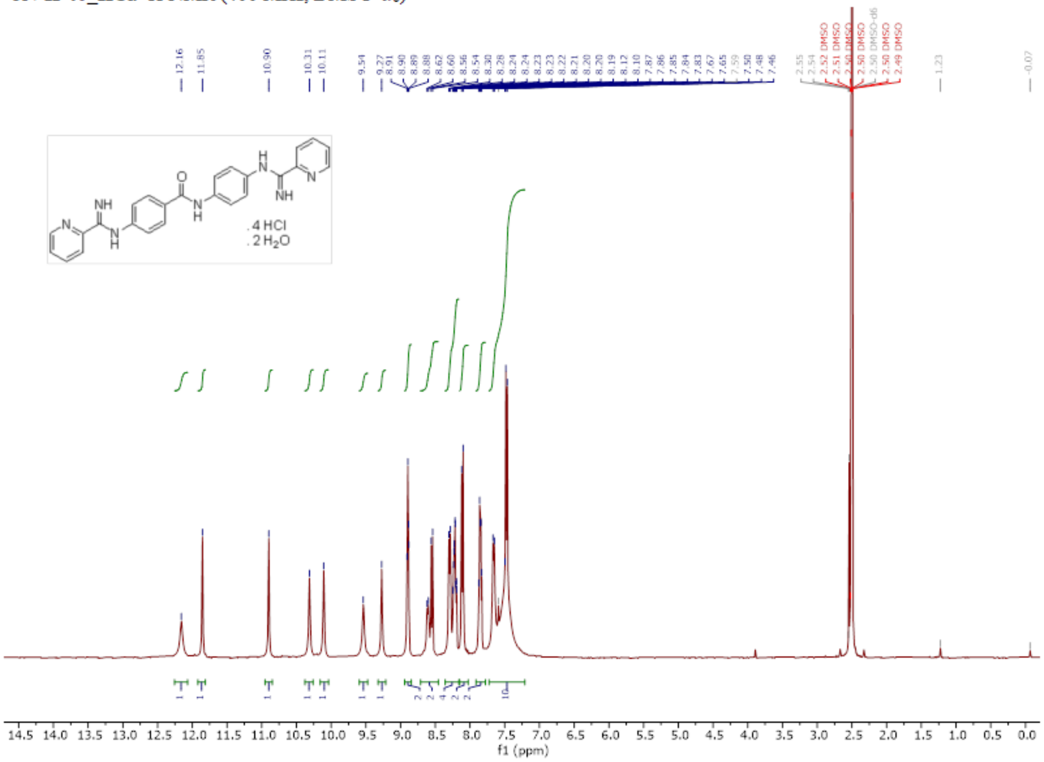

Supplement Figure 2. ^{1}H NMR spectra and HPLC-MS traces of JN-II40_HCl

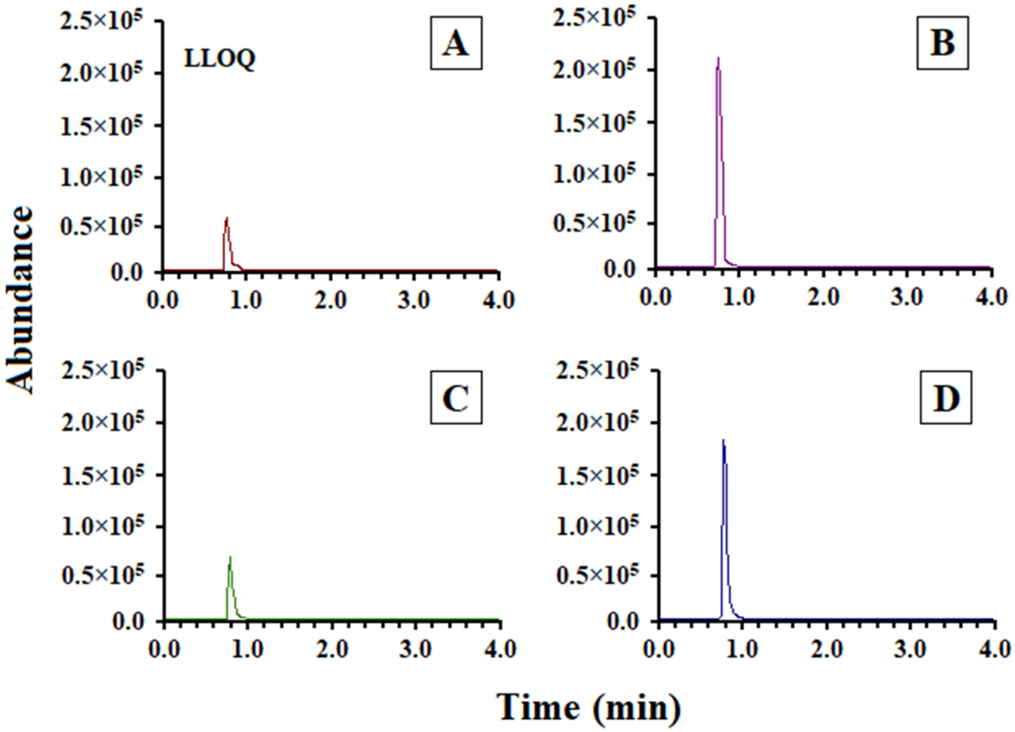

Supplement Figure 3. Representative HPLC-MS/MS chromatograms of JNII40 showing **(a)** standard sample at 15 ng/mL (LLOQ), **(b)** standard sample at 150 ng/mL, **(c)** plasma sample at low concentration and **(d)** plasma sample at medium concentration.
